# Bcl3 Deficiency Leads to Hyperinflammation in Zebrafish

**DOI:** 10.3390/cells14241935

**Published:** 2025-12-05

**Authors:** Chengjian Fan, Nana Ai, Wei Ge, Vivien Ya-Fan Wang

**Affiliations:** 1 Department of Biomedical Sciences, Faculty of Health Sciences, University of Macau, Avenida da Universidade, Taipa, Macau SAR, China; yc07649@um.edu.mo (C.F.); nanaai@um.edu.mo (N.A.); weige@um.edu.mo (W.G.); 2 Centre of Reproduction, Development and Aging (CRDA), Faculty of Health Sciences, University of Macau, Avenida da Universidade, Taipa, Macau SAR, China; 3 Cancer Centre, Faculty of Health Sciences, University of Macau, Avenida da Universidade, Taipa, Macau SAR, China; 4 MoE Frontiers Science Center for Precision Oncology, University of Macau, Avenida da Universidade, Taipa, Macau SAR, China

**Keywords:** Bcl3, NF-κB signaling, inflammation, infection, zebrafish model

## Abstract

**Highlights:**

**What are the main findings?**

- The establishment of zebrafish as a vertebrate model for dissecting Bcl3 mediated immune reaction.
- Anti-inflammatory treatment effectively alleviated inflammation and improved survival.

**What are the implications of the main findings?**

- Bcl3 functions as a key negative regulator of immune activation in vivo.

**Abstract:**

B-cell leukemia/lymphoma protein 3 (Bcl3), a member of the IκB family proteins, regulates the transcriptional activities of the NF-κB family of transcription factors. It is known that aberrant Bcl3 activities induce malignancies of both hematologic and non-hematologic origins. Overexpressed, mutated and/or phosphorylated Bcl3 has been implicated in several cancers due to its altered transcriptional activities. However, the physiological function of Bcl3 in immune homeostasis remained elusive to date. In this study, Bcl3 knockout zebrafish were generated to investigate its role in immune regulation. Bcl3 deficient zebrafish exhibited growth retardation and significantly reduced survival. Histological analyses revealed the absence of Hassall bodies in the thymus and hepatocellular nuclear abnormalities, indicating compromised integrity of the immune organs. Zebrafish with Bcl3 deficiency further showed enhanced immune responses and increased susceptibility to both bacterial and viral infections, resulting in significantly elevated levels of pro-inflammatory cytokines *il1b*, *il6*, *il8*, and *tnfa*. Treatment with the anti-inflammatory drug dexamethasone (Dex) effectively alleviated inflammation, downregulated pro-inflammatory cytokine expressions and improved survival. Collectively, our findings demonstrate Bcl3 as a key regulator of immune activation in vivo, highlighting its role in maintaining immune homeostasis and promoting organismal survival.

## 1. Introduction

Bcl3 was first identified as a proto-oncogene in human B-cell chronic lymphocytic leukemia with t(14;19)(q32;q13) chromosomal translocation [1]. Subsequent studies demonstrated that Bcl3 promotes cellular differentiation and proliferation [2,3]. Aberrant Bcl3 expression and post-translational modification have been implicated in the development of both hematologic and solid malignancies. In solid tumors, Bcl3 overexpression or phosphorylation enhances tumor growth, invasion, and survival [4,5,6].

Bcl3 belongs to the inhibitor of nuclear factor kappa B (NF-κB) (IκB) family which regulates the transcriptional activities of NF-κB. Unlike classical IκB proteins, overexpressed Bcl3 predominantly resides in the nucleus and interacts only with the p50 and p52 homodimers within the NF-κB family [7,8,9,10,11,12,13]. Bcl3 functions to recruit either co-activators, such as Tip60, or co-repressors, such as HDAC3, and fine-tune the target gene expression depending on cellular context [14,15,16,17]. This dual regulatory potential positions Bcl3 as a critical co-regulator within the NF-κB signaling network.

Beyond its oncogenic potential, Bcl3 has also been shown to be a critical regulator of immune and inflammatory processes. In macrophages, Bcl3 suppresses the lipopolysaccharide (LPS)-induced transcription of pro-inflammatory genes such as TNFα and IL6, thereby preventing excessive inflammation [18,19]. Conversely, hepatocyte-specific overexpression of Bcl3 exacerbates non-alcoholic steatohepatitis (NASH) by promoting hepatic steatosis, inflammation, and insulin resistance [20]. Mouse knockout models have revealed that *bcl3* deficiency leads to germinal center defects and impaired T cell-dependent antibody responses [21]. However, most existing evidence was derived from cancer cell lines, inflammatory disease models, or limited tissue-specific studies in mammals, rather than from healthy organisms under physiological conditions. As a result, the systemic, organism-level role of Bcl3 in coordinating immune homeostasis, inflammation control, and survival remains largely unexplored. This gap underscores the need for a genetically tractable vertebrate model to dissect the physiological function of Bcl3 under normal and immune-challenged conditions.

The zebrafish (*Danio rerio*) has emerged as a powerful model in biological research, and it has been successfully used to understand the biological activities of gene orthologs related to those that cause human genetic diseases [22,23]. A comparison to the human genome shows ~71% of human genes have at least one zebrafish ortholog [23,24]. All five mammalian NF-κB family members: RelA/p65, RelB, c-Rel, NF-κB1/p105 (p50), and NF-κB2/p100 (p52), have been identified in zebrafish (Table S1) [25,26,27]. Most IκB family members have also been identified or predicted in zebrafish, including IκBα (IκBαa and IκBαb in zebrafish), -β, -ε, -γ, -δ, and Bcl3 [25,28,29], supporting an essential and conserved regulatory network for NF-κB signaling from fish to mammal. The zebrafish *bcl3* gene was identified and sequenced in 2006, and it shows conserved domain organization as the mammalian protein (ZFIN ID: ZDB-GENE-061013-1); however, no functional study of zebrafish Bcl3 has been reported so far. In this study, we generated Bcl3 knockout zebrafish to investigate the physiological function of Bcl3 in immune regulation. Comprehensive phenotypic, histological, and transcriptomic analyses were conducted to assess the development, immune organ integrity, and transcriptional responses upon Bcl3 deficiency. Zebrafish with Bcl3 deficiency exhibited growth retardation, reduced survival, compromised integrity of immune organs, enhanced pro-inflammatory responses, and increased susceptibility to both bacterial and viral infections. Treatment with anti-inflammatory drugs effectively alleviated inflammation, downregulated pro-inflammatory cytokine expressions, and improved survival. Collectively, our study established zebrafish as a powerful vertebrate model for dissecting Bcl3 mediated immune regulation. Our findings provided new insights into Bcl3′s function as a key negative regulator of immune activation in vivo, emphasizing its importance in maintaining immune homeostasis and promoting organismal survival.

## 2. Materials and Methods

### 2.1. Ethics Statement

All experimental procedures involving zebrafish (*Danio rerio*) were managed by the Animal Protection Act, enacted by the Legislative Council of the Macao Special Administrative Region under Article 71(1) of the Basic Law. The animal study protocol was approved by the University of Macau’s Research Ethics Panel (approval code UMAEC-027-2015 in 2015).

### 2.2. Zebrafish Maintenance

WT zebrafish of the AB strain were used in this study to generate *bcl3* mutant lines. Zebrafish larvae were maintained in static water from 0 to 5 days post-fertilization (dpf) without feeding, fed with egg yolk and paramecium between 5 and 10 dpf, and with *Artemia* from 10 to 20 dpf. The juveniles were then transferred to the ZebTEC aquarium system (Tecniplast, Buguggiate, Italy) and maintained at 28 °C, pH 7.5, with a photoperiod of 14 h of light (8:00 a.m. to 10:00 p.m.) and 10 h of dark. They were fed the Otohime fish diet (Marubeni Nissin Feed, Tokyo, Japan) delivered by the Tritone automatic feeding system (Tecniplast). The test zebrafish used in this study were mixed sex.

### 2.3. Bcl3 Knockout Generation

The zebrafish *bcl3* genomic and coding sequences were obtained from Ensembl (asia.ensembl.org/Danio rerio) under accession number ENSDART00000130641.3. The *bcl3* mutant lines were generated by CRISPR/Cas9-mediated mutagenesis according to a previously reported case [30,31]. Primer pairs for CRISPR/Cas9 knockout are shown in Table S3. Single guide RNAs (sgRNAs) were transcribed in vitro using the mMACHINE T7 kit (Invitrogen, Waltham, MA, USA). Purified sgRNA (50 ng/μL) were co-microinjected with Cas9 protein (20 uM) (NEB #M0646) into one-cell or two-cell stage embryos, which were injected with 4.6 nL/embryo using a Drummond Nanoject syringe (Drummond Scientific, Broomall, PA, USA). DNA was extracted from individual embryos 24 h post-fertilization (hpf), followed by high-resolution melting curve analysis (HRMA), and heteroduplex migration analysis (HMA) of the injected embryos. Approximately 70% of the F0 generation embryos showed successful genome editing, and the remaining edited embryos were raised to adulthood as potential F0 generation founders. Adult F0 mosaic founders were crossed with wild-type (WT) zebrafish to obtain F1 offspring. Since F1 fish typically carry multiple mutation types, F1 juveniles were raised to 2 months of age, and caudal fin clips were collected for genomic DNA extraction. PCR products were cloned into plasmids and Sanger-sequenced to identify the exact indel patterns. Male and female F1 individuals carrying the same mutation type were selected and paired to generate F2 progeny. As expected under Mendelian segregation, the F2 generation consisted of approximately 25% *bcl3^−/−^*, 50% *bcl3^+/−^*, and 25% *bcl3^+/+^*. Genotypes of F2 larvae were confirmed using HRMA, HMA, and sequencing. F2 homozygous *bcl3^−/−^* fish displayed stable germline transmission of the mutation.

### 2.4. DNA Extraction and Genotyping

Genomic DNA was extracted from embryos or caudal fin pieces by alkaline lysis. Genotyping of early embryos was performed on individual embryos using whole-embryo DNA extraction. Caudal fin tissue for genomic DNA extraction was collected from adult zebrafish at 2–3 months of age. The samples were incubated in 40 μL of 50 mM NaOH at 95 °C for 10 min, followed by adding 4 μL Tris-HCl (pH 8.0) for neutralization. The supernatant was obtained by centrifugation at 14,000× *g* for 5 min and used directly as the template for HRMA. Melting curves were analyzed using Precision Melting Analysis software (Bio-Rad, Hercules, CA, USA). The primers used for genotyping are listed in Table S3.

### 2.5. Calculation of Body Surface Area (BSA) and Body Mass Index (BMI)

Body surface area (BSA) was estimated using the following formula, validated for fish of similar size and shape to zebrafish:

$${BSA(cm}^{2})={8.46\times (body\;weight\left(g\right))}^{0.66}$$

Body mass index (BMI) was calculated as

$$BMI=\frac{body\;weight\left(g\right)}{(Body\;length{\left(cm)\right)}^{2}}$$

### 2.6. Survival Analysis

The survival rate from 0 to 30 dpf was examined by calculating the ratio of genotypes obtained from crosses between *bcl3*^+/−^ zebrafish. To evaluate long-term survival (30–150 dpf), offspring from WT X WT zebrafish, *bcl3^−/−^* X *bcl3^−/−^* zebrafish were monitored. After the offspring reached 30 dpf, they were transferred to the recirculating aquarium system, and mortality was recorded daily until 150 dpf.

### 2.7. Food Intake Assay

An external calibration curve was first established to determine the relationship between the *Artemia* number and optical density (OD) [32]. Groups of six zebrafish per tank were fasted for 24 h in 1.5 L of system water, while a tank without fish served as the feeding control. Each tank was fed with 1 mL of freshly prepared *Artemia* suspension for 15 min; then the fish were transferred to new tanks through a fish net. The fish net mesh was sufficiently wide to allow *Artemia* to pass through unimpeded. The remaining *Artemia* were then quantified by filtration through a 100 μm filter and finally resuspended in 1 mL of system water for OD determination. The number of *Artemia* consumed was calculated using the calibration curve. Only *Artemia* was used during the feeding assay, and no flake diet was provided during the analysis period.

$$Number\;of\;Artemia\;consumed=N_{fish-free\;tank}\;{-\;N}_{remaining\;after\;feeding}$$

$$Normalized\;food\;intake=\frac{Number\;of\;Artemia\;consumed}{Fish\;body\;weight\;(mg)}$$

### 2.8. Motility Assay

Zebrafish swimming behavior was recorded and analyzed using a DanioVision monitoring chamber connected to EthoVision XT 14 video tracking software (Noldus, Wageningen, The Netherlands). Swimming behavior was monitored using an infrared automatic video tracking system. Individual fish were placed in a 10 cm Petri dish for behavioral recording. All assays were conducted at 28 ± 1 °C. Each fish was monitored for 15 min, including an initial 5 min acclimation period. The tracking software automatically recorded and analyzed the movement trajectory, total distance traveled, and average swimming speed.

### 2.9. Hematoxylin and Eosin (H&E) Staining

Fish were fixed in Bouin’s solution for at least 5 days and then dehydrated and paraffin-embedded on an ASP6025S automated vacuum tissue processor (Leica, Wetzlar, Germany). Specimens were sectioned at 5 μm using a Leica microtome (Leica). After deparaffinization, hydration, and H&E (Kewdale, WA, Australia) staining, sections were observed using a Nikon ECLIPSE Ni-U microscope, and micrographs were captured with a Digit Sight DS-Fi2 digital camera (Nikon, Shinagawa, Japan).

### 2.10. RNA Extraction and RT-qPCR

Total RNA was extracted from the zebrafish tissues using TRIzol reagent (Invitrogen). A total of 200 μL of TRIzol was added to each 1.5 mL microcentrifuge tube containing homogenized zebrafish tissue using an electric grinder. After adding 50 μL of chloroform, the samples were vortexed for 2 min and incubated at room temperature for 8 min. The aqueous phase (~80–100 μL) was transferred to a new tube, mixed with 100 μL of isopropanol, and incubated at −20 °C for 2 h to precipitate RNA. The samples were centrifuged at 20,000× *g* for 10 min at 4 °C, and the RNA pellet was washed with 300 μL of 75% ethanol at 7500× *g* for 5 min. After removing residual ethanol, the RNA was dissolved in 10 μL of DEPC-treated water. RNA concentration and purity were measured using a NanoDrop spectrophotometer (Thermo Scientific, Waltham, MA, USA).

First-strand cDNA was synthesized from 1 μg of total RNA using the PrimeScript™ II 1st Strand cDNA Synthesis Kit (Takara, Kusatsu, Shiga, Japan). RT-qPCR was performed on a CFX96 RT-qPCR System (Bio-Rad) using SsoFast EvaGreen Supermix (Bio-Rad). Primers are listed in Table S3. Melting curve analysis confirmed the primer specificity, and all reactions were conducted in duplicate to ensure accuracy. Target-gene expression levels were normalized to *ef1a* and calculated relative to the corresponding control group.

### 2.11. RNA Sequencing (RNA-seq)

Liver, spleen, and muscle tissues were collected from 3-month-old *bcl3^+/+^* and *bcl3^−/−^* zebrafish for RNA-seq. Three biological replicates were prepared per tissue type, with each replicate consisting of pooled tissues from three individual zebrafish. The samples were sent to Novogene (Novogene, Beijing, China) for total RNA extraction, library preparation, and next-generation sequencing.

### 2.12. LPS and Poly(I:C) Injection

LPS and poly(I:C) injection were performed using offspring of WT × WT and *bcl3^−/−^* × *bcl3^−/−^* crosses. At 4 dpf, 6 nL of 0.1 mg/mL LPS (*E. coli* O55:B5, Sigma, St. Louis, MO, USA) or 2 mg/mL poly(I:C) (Sigma) was injected into the yolk of larvae, with approximately 100 larvae per each group. Following injection, larvae were maintained at 28.5 °C and monitored for signs of inflammation and mortality over the subsequent 24 h.

### 2.13. Immersion of LPS-Injected Larvae in Dex Solution

At 4 dpf, WT and *bcl3^−/−^* zebrafish larvae were injected with 13.8 nL of 0.1 mg/mL LPS (*E. coli* O55:B5, Sigma). One h after injection, larvae were immersed in a dexamethasone (Dex) (Sigma) solution prepared by diluting a 30 mg/mL stock in DMSO to a final concentration of 0.3 mg/mL in 2 mL of system water. An equivalent volume of DMSO was used as the control. Survival rates were recorded at 24, 48, and 72 h post-injection. At 48 h post-LPS injection, a subset of larvae from each treatment group was collected for total RNA extraction and gene expression analysis.

### 2.14. Dex Injections in WT and bcl3^−/−^ Zebrafish

At 35 dpf, WT and *bcl3^−/−^* zebrafish were subjected to repeated injections of Dex (Sigma) or DMSO as a control every three days until 138 dpf via intraperitoneal injection. Dex was prepared as a 0.15 mg/mL solution in DMSO. The zebrafish were injected with 26.8 nL Dex solution or DMSO.

### 2.15. Statistical Analysis

All analyses were performed using GraphPad Prism 6.0. Data are shown as mean ± standard deviation (SD). Survival curves were compared using the log-rank test. For ≥3 groups, normally distributed data were analyzed by one-way ANOVA with Tukey’s post hoc test; non-normally distributed data were analyzed using the Kruskal–Wallis test with Holm-corrected Mann–Whitney U comparisons. Data normality was evaluated using the Shapiro–Wilk test. Two-group comparisons were performed using Student’s *t*-test. Binary variables were analyzed using chi-square tests (see Table S2). Significance was defined as *p* < 0.05.

## 3. Results

### 3.1. Bcl3 Deficiency Leads to Reduced Growth and Survival in Zebrafish

To investigate the physiological role of Bcl3 in zebrafish, *bcl3^−/−^* zebrafish were generated using the CRISPR/Cas9 system. A single guide RNA (sgRNA) was designed to target the exon 2 of zebrafish *bcl3* gene (Figure S1A, top) which generated three different deletion mutations, lacking 10-, 5-, and 7-base pairs (bp), respectively, in the *bcl3* gene, with the same phenotype. Analyses of the 4-month genotype ratio of these *bcl3^−/−^* zebrafish revealed that the proportion of *bcl3^−/−^* zebrafish (~10%) was considerably lower than the expected Mendelian ratio (25%) in all three deletion mutants (Figure S1A, bottom). The 10 bp deletion mutant zebrafish with the highest survival rate were used for all subsequent studies. This 10 bp deletion introduced a premature stop codon in the Bcl3 protein (Figure S1B); the high-resolution melting analysis (HRMA) and the heteroduplex mobility assay (HMA) revealed distinct genotypes, *bcl3^+/+^*, *bcl3^+/−^*, and *bcl3^−/−^* (Figure S1C,D). The mRNA expression levels of NF-κB subunits *rela*, *relb*, *rel*, *nfkb1*, and *nfkb2* were examined using RT-qPCR upon bcl3 knockout. All five NF-κB subunits showed comparable expression levels in the liver, spleen, and muscle samples from adult *bcl3^+/+^* and *bcl3^−/−^* fish at 4 months old (Figure S2). These findings suggested that the reduced growth and survival of *bcl3^−/−^* zebrafish were not due to the changes in NF-κB subunits’ expression.

**Figure 1:**
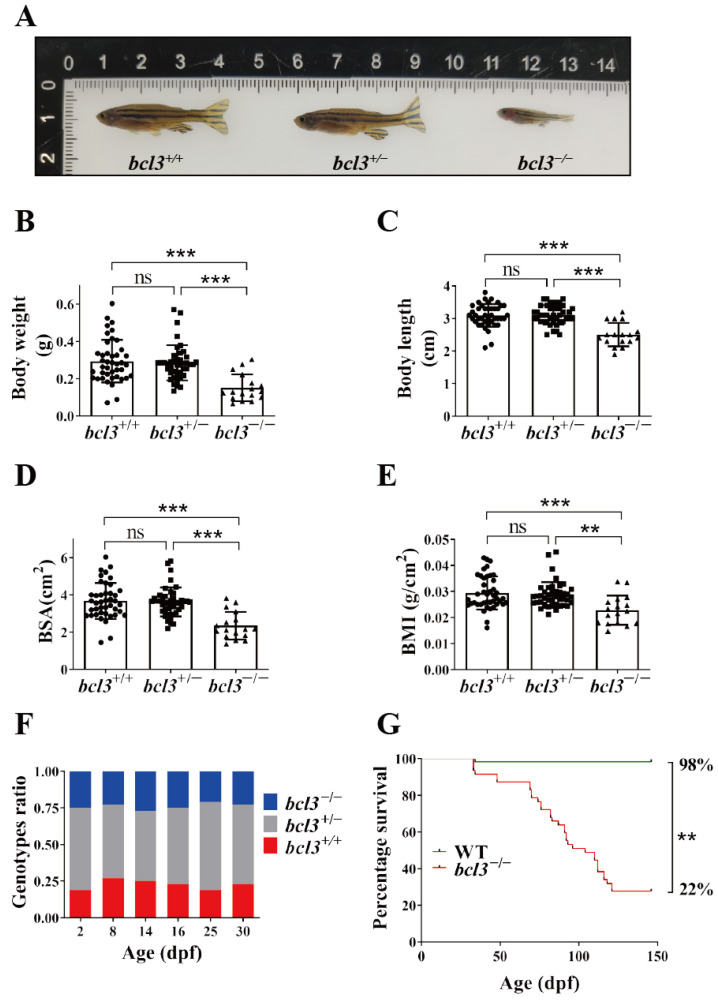
Phenotypes of *bcl3^−/−^* zebrafish. (**A**) Representative images of *bcl3^+/^*^+^, *bcl3^+/^*^−^, and *bcl3^−/^*^−^ zebrafish at 4 months old. (**B**) Body weight, (**C**) body length, (**D**) body surface area (BSA), and (**E**) body mass index (BMI) of *bcl3^+/^*^+^, *bcl3^+/^*^−^, and *bcl3^−/^*^−^ zebrafish. BSA was defined by: BSA (cm^2^) = 8.46 × (body weight (g))^0.66^; BMI was calculated as body weight (g) divided by body length square (cm^2^). Body length data were normally distributed and analyzed using one-way ANOVA followed by Tukey’s post hoc test; whereas body weight data, BSA, and BMI values did not meet normality assumptions and were analyzed using the Kruskal–Wallis test with Holm-corrected Mann–Whitney U post hoc comparisons. (**F**) Genotype ratio of *bcl3^+/^*^+^, *bcl3^+/^*^−^, and *bcl3^−/^*^−^ larvae at indicated dpf. At each time point, 48 larvae were genotyped. (**G**) Survival curves of zebrafish WT and *bcl3^−/^*^−^ from 35 to 150 dpf, analyzed using the log-rank (Mantel–Cox) test. *** *p* < 0.001, ** *p* < 0.01, ns indicates not significant. Error bars represent standard deviation (SD).

In addition to having a low survival rate, *bcl3*^−/−^ zebrafish were also found to be significantly smaller than both *bcl3^+/+^* and *bcl3^+/−^* zebrafish with markedly reduced standard body weight, body length, body surface area (BSA), and body mass index (BMI) (Figure 1A–E). Quantitative measurements confirmed that only homozygous mutants showed significantly reduced standard body length and body weight, whereas *bcl3^+/−^* fish displayed body size comparable to *bcl3^+/+^* controls. To determine the onset of mortality, the genotype ratio of *bcl3^+/+^*, *bcl3^+/−^*, and *bcl3^−/−^* larvae was examined at 2, 8, 14, 16, 25, and 30 dpf. The proportion of *bcl3^−/−^* larvae remained consistent with Mendelian expectations up to 30 dpf (Figure 1F), which suggested that lethality occurred during the juvenile or young adult stages of *bcl3* deficient zebrafish. The survival of *bcl3^−/−^* zebrafish was further monitored from 30 to 150 dpf in comparison to WT zebrafish. The progressive mortality *bcl3^−/−^* zebrafish started from ~70 dpf. The survival rate of *bcl3^−/−^* mutant fish sharply declined compared with the WT offspring, with an overall survival rate of 22%, significantly lower than the 98% survival rate of WT zebrafish (Figure 1G). Together, these results demonstrated that *bcl3* deficiency causes growth retardation and post-juvenile lethality in zebrafish, underscoring the essential role of Bcl3 in development and organismal survival.

### 3.2. Bcl3 Deficiency Impairs Feeding Behavior and Motility in Zebrafish

**Figure 2:**
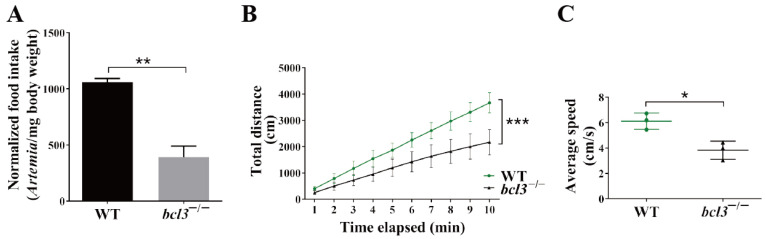
Feeding behavior and motility of *bcl3^−/−^* zebrafish. (**A**) Normalized food intake (number of *Artemia* per milligram body weight) of WT and *bcl3^−/^*^−^ zebrafish at 4 months old. (**B**) Total movement distance and (**C**) average movement speed of WT and *bcl3^−/^*^−^ zebrafish recorded over 10 min. *** *p* < 0.001, ** *p* < 0.01, * *p* < 0.05 (*t*-test). Error bars represent SD.

We speculated that the reduced size and weight of *bcl3^−/^*^−^ zebrafish was a result of insufficient feeding; therefore, the feeding behavior of *bcl3^−/^*^−^ zebrafish at 4 months old was monitored. An *Artemia* (also known as brine shrimp) feeding assay was performed. An external standard curve was first generated to correlate the number of *Artemia* with optical density (OD) (Figure S3). The feeding assays showed that *bcl3^−/−^* zebrafish consumed significantly lower amount of *Artemia* compared to the WT zebrafish (Figure 2A), suggesting a marked reduction in feeding behavior.

In addition, zebrafish motility was also assessed by tracking their swimming behavior. The *bcl3^−/−^* zebrafish at 4 months old were also found to exhibit significantly reduced motility, as evidenced by reduced swimming distances as well as swimming speeds (Figure 2B,C). Both the reduced food intake and motility observed in *bcl3^−/−^* fish likely contributed to their growth retardation and low survival.

### 3.3. Loss of Bcl3 Compromises Immune Organ Integrity and Immune Cell Function

**Figure 3:**
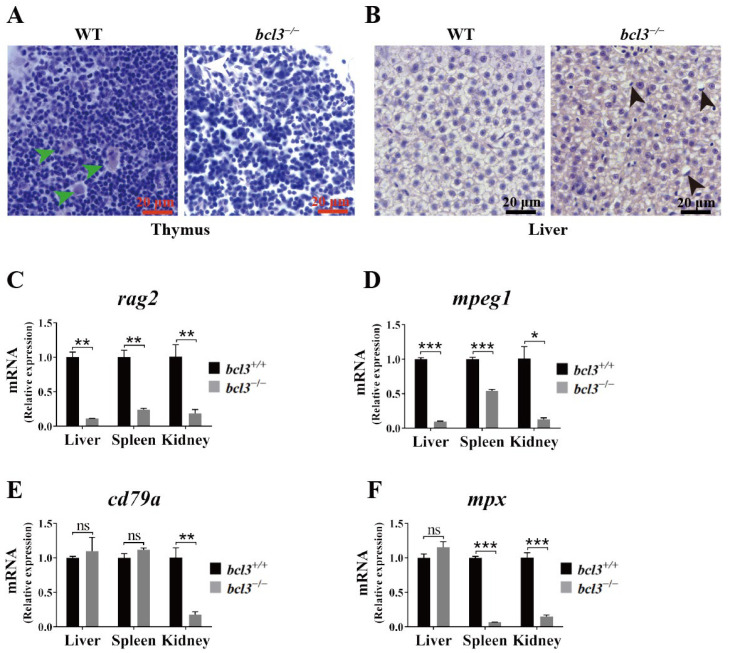
Histological abnormalities and reduced immune cell marker expression in *bcl3^−/−^* zebrafish. (**A**) H&E-stained sections of thymus from WT and *bcl3^−/^*^−^ zebrafish at 4 months of age. Green arrows indicate Hassall bodies. (**B**) H&E-stained sections of liver from WT and *bcl3^−/^*^−^ zebrafish. Black arrows indicate hepatocytes with nuclear displacement and pyknosis. Relative mRNA expression of (**C**) *rag2* (T cell marker), (**D**) *mpeg1* (macrophage marker), (**E**) *cd79a* (B cell marker), and (**F**) *mpx* (neutrophil marker) in the liver, spleen, and kidney of *bcl3^+/+^* and *bcl3^−/−^* zebrafish at 4 months of age. *** *p* < 0.001, ** *p* < 0.01, * *p* < 0.05 (*t*-test), ns indicates not significant. Error bars represent SD.

Bcl3 was known to function together with the NF-κB family of transcription factors, a key regulator of immune responses. Therefore, histological analyses were performed on the key immune organs, the thymus and liver, in zebrafish at 4 months old. Hematoxylin and eosin (H&E) staining revealed the absence of Hassall bodies in the thymus of *bcl3^−/−^* zebrafish (Figure 3A). Hassall’s functions include the removal of apoptotic thymocytes, the maturation of developing thymocytes, and the direction of dendritic cells to induce CD4^+^CD25^+^ regulatory T cells in the human thymus [33]. Additionally, nuclear displacement and pyknosis of hepatocytes were observed in the liver of *bcl3^−/−^* zebrafish (Figure 3B), indicating structural disruption consistent with impaired immune or metabolic activities. To further investigate the impact of *bcl3* deficiency on immune function, the mRNA level of immune cell marker genes was checked by RT-qPCR. The expression of T cell marker *rag2* and macrophage marker *mpeg1* was found to be significantly lower in the liver, spleen, and kidney of *bcl3^−/−^* zebrafish (Figure 3C,D); the expression of B cell marker *cd79a* was significantly reduced in the kidney (Figure 3E); and the expression of neutrophil marker *mpx* was significantly reduced in both the spleen and kidney (Figure 3F). The combination of histological abnormalities in immune organs and the reduced expression of immune cell markers strongly suggested that *bcl3* deficiency compromises the immune organ integrity and impairs immune cell development.

### 3.4. Transcriptomic Analyses Reveals Systemic Immune Hyperactivation in bcl3 Deficient Zebrafish

To further evaluate the immune dysregulation in adult *bcl3^−/−^* zebrafish, RNA sequencing (RNA-seq) was performed using liver, spleen, and skeletal muscle samples of 3-month-old zebrafish under basal conditions. The 3-month stage (~90 dpf) was selected since it represents the midpoint of the progressive mortality period of *bcl3^−/−^* fish (Figure 1G), and it also coincides with full sexual maturity. Tissue selections were guided by their biological roles: the liver serves as an immune–metabolic hub and principal source of acute-phase and interferon-stimulated programs in fish; the spleen is a major lymphoid/hematopoietic organ in teleosts; and the skeletal muscle is a large peripheral tissue sensitive to circulating cytokines, which enables the assessment of systemic spillover of inflammatory and antiviral signaling.

**Figure 4:**
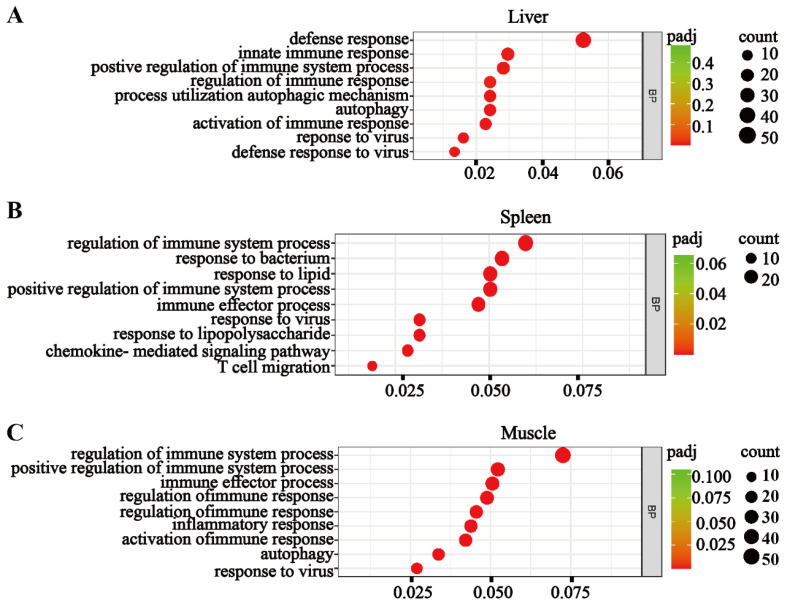
Transcriptomic analyses reveal systemic immune activation in *bcl3^−/−^* zebrafish. Gene Ontology (GO) enrichment maps showing upregulated biological processes in the (**A**) liver, (**B**) spleen, and (**C**) muscle of *bcl3^−/−^* zebrafish compared with *bcl3^+/+^* controls. The *y*-axis lists pathway terms, and the *x*-axis represents gene ratios. Circle size indicates the number of genes, while color intensity denotes the adjusted *p*-value (padj).

The RNA-seq analyses revealed significantly upregulated immune responses in all three tissues of *bcl3^−/−^* compared to the *bcl3^+/+^* zebrafish control (Figure 4). Gene Ontology (GO) enrichment analyses showed an increased expression of various cytokines and chemokines, which are critical mediators of inflammation responses, as well as antiviral immune responses. In addition, key factors in the JAK/STAT signaling pathway were found to be upregulated in the liver and muscle of *bcl3^−/−^* zebrafish (Figure S4), suggesting that the elevated immune response may be mediated through the JAK/STAT signaling pathway in the absence of Bcl3. Collectively, these transcriptomic results suggested that *bcl3* deficiency in zebrafish causes systemic immune hyperactivation even under basal conditions, leading to chronic inflammation and upregulations of antiviral genes in adult zebrafish. This finding supports the notion that Bcl3 is an essential regulator for maintaining immune equilibrium in the mature fish.

### 3.5. Bcl3 Deficiency Exacerbates Inflammatory and Antiviral Responses to LPS and Poly(I:C) Stimulation

The immune response is essential for protecting an organism from external damage; however, excessive immune activation may cause tissue damage and adversely affect survival. We speculated that the reduced survival rate of *bcl3^−/−^* zebrafish is due to an overactive immune response. To test this, we evaluated Bcl3′s function upon immune challenge using zebrafish larvae. Zebrafish larvae have an immature immune system; at 4 dpf, zebrafish larvae rely exclusively on innate immunity, whereas adaptive components develop 4–6 weeks later [34,35,36]. Therefore, basal inflammatory activity is minimal at the larval stage and *bcl3*^−/−^ larvae exhibited normal baseline viability (Figure S5). *bcl3^−/−^* larvae were challenged using either bacterial lipopolysaccharide (LPS) or viral mimetic poly(I:C). In fish, LPS triggers MyD88-independent NF-κB signaling to drive the expression of pro-inflammatory cytokines and antiviral genes [37]; while poly(I:C) engages RIG-I-like receptor (RLR)-NF-κB pathways to induce combined pro-inflammatory and type I interferon responses [38].

**Figure 5:**
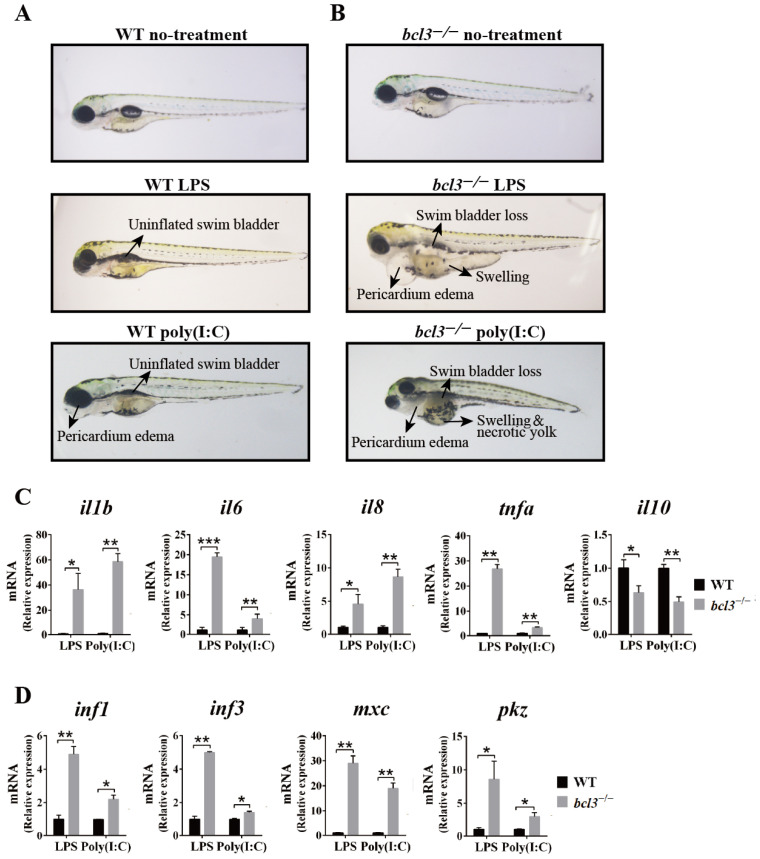
Immune responses upon LPS and poly(I:C) stimulation in *bcl3^−/−^* larvae. Representative morphology of (**A**) WT and (**B**) *bcl3^−/−^* larvae under untreated condition or at 24 h upon LPS or poly(I:C) injection. Relative mRNA expression of (**C**) pro-inflammatory genes *il1b*, *il6*, *il8*, and *tnfa* and anti-inflammatory gene *il10*, and (**D**) antiviral genes *ifn1*, *ifn3*, *mxc*, and *pkz* in WT and *bcl3^−/^*^−^ larvae 24 h upon LPS or poly(I:C) injection. The data was analyzed from three independent experiments performed in triplicate. *** *p* < 0.001, ** *p* < 0.01, * *p* < 0.05 (*t*-test). Error bars represent SD.

Upon stimulation with LPS or poly(I:C), WT larvae displayed moderate symptoms, such as an uninflated swim bladder and slight pericardial edema compared to non-stimulated fish (Figure 5A). In contrast, *bcl3^−/−^* zebrafish showed severe symptoms, including the complete disappearance of the swim bladder, pronounced pericardial edema, swelling, and yolk necrosis (Figure 5B). Quantitative analysis of larvae phenotypes further showed the increased mortality and incidence of inflammatory abnormalities of *bcl3^−/−^* larvae compared to WT controls (Table S2). These results demonstrated the intensified immune sensitivity of *bcl3^−/−^* zebrafish to pathogenic stimuli. RT-qPCR analyses further showed that the pro-inflammatory cytokines, *il1b*, *il6*, *il8*, and *tnfa*, were upregulated; while anti-inflammatory gene *il10* was downregulated in *bcl3^−/−^* larvae upon LPS and poly(I:C) stimulation (Figure 5C). Antiviral genes *ifn1*, *ifn3*, *mxc*, and *pkz* were also significantly upregulated in *bcl3^−/−^* larvae (Figure 5D). These results indicated that the absence of Bcl3 leads to an overactive immune response, which may contribute to the reduced survival of *bcl3^−/−^* zebrafish due to excessive inflammation and associated physiological damages.

### 3.6. Anti-Inflammatory Treatment Improves the Survival of bcl3^−/−^ Zebrafish

**Figure 6:**
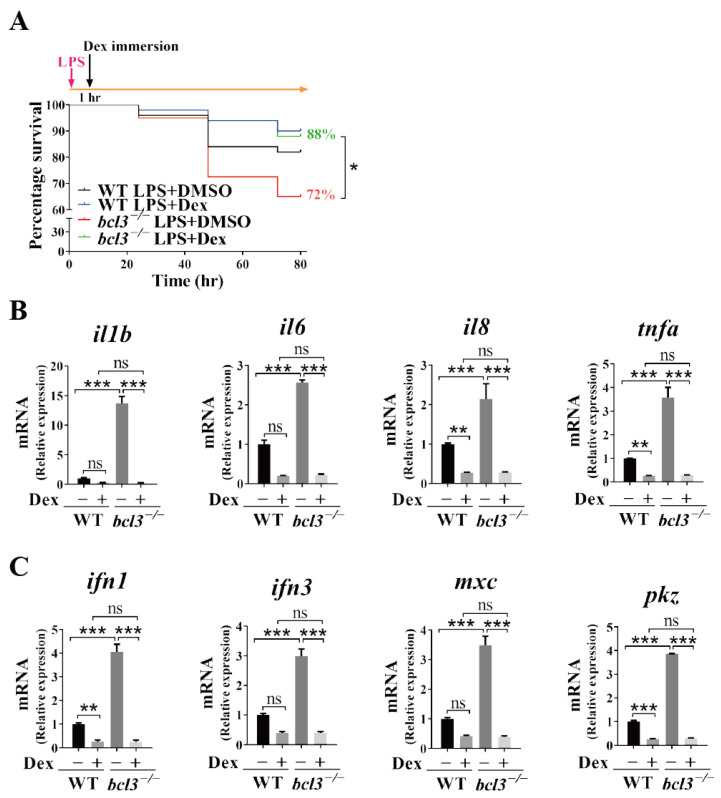
Effects of Dex treatment in *bcl3^−/−^* larvae. (**A**) Survival curve of WT and *bcl3^−/^*^−^ larvae upon LPS injection followed by Dex immersion, analyzed using the log-rank (Mantel–Cox) test. Relative mRNA expression of (**B**) pro-inflammatory genes *il1b*, *il6*, *il8*, and *tnfa*, and (**C**) antiviral genes *ifn1*, *ifn3*, *mxc*, and *pkz* in WT and *bcl3^−/^*^−^ larvae at 48 h upon LPS injection followed by Dex immersion. The data was analyzed from three independent experiments performed in triplicate. Statistical significance was assessed using one-way ANOVA followed by Tukey’s post hoc test. *** *p* < 0.001, ** *p* < 0.01, * *p* < 0.05, ns indicates not significant. Error bars represent SD.

We further evaluated the effects of the anti-inflammatory drug treatment, Dex, on the attenuation of the hyperinflammation observed upon *bcl3* deficiency in both larvae and adult fish. At 4 dpf, both WT and *bcl3^−/−^* larvae were first injected with LPS followed by Dex immersion at 1 h post-injection; the mortality rate was then monitored at 24, 48, and 72 h post-treatment and total RNA was extracted at 48 h for RT-qPCR analyses. Dex treatment significantly improved the survival rate of *bcl3^−/−^* larvae compared with DMSO control (Figure 6A). mRNA levels of both pro-inflammatory cytokines *il1b*, *il6*, *il8*, and *tnfa* (Figure 6B), and antiviral genes *ifn1*, *ifn3*, *mxc*, and *pkz* (Figure 6C) were significantly reduced upon the treatment of Dex. No significant differences were observed in these genes between Dex-treated WT and *bcl3^−/−^* larvae, indicating that the treatment of the anti-inflammation drug restored the hyperinflammation in *bcl3^−/−^* larvae and improved survival.

**Figure 7:**
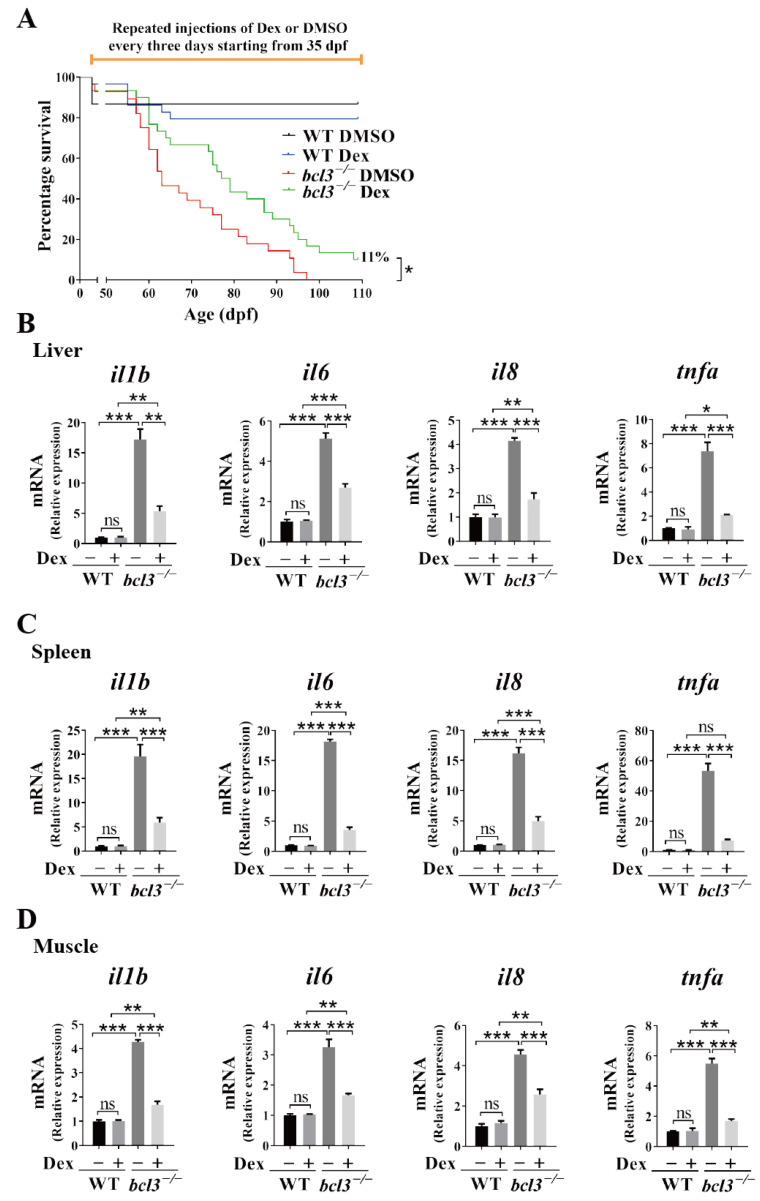
Effects of Dex treatment in adult *bcl3^−/−^* zebrafish. (**A**) Survival curve of WT and *bcl3^−/^*^−^ zebrafish with Dex injection, analyzed using the log-rank (Mantel–Cox) test. Relative mRNA expression of pro-inflammatory genes *il1b*, *il6*, *il8*, and *tnfa* in (**B**) liver, (**C**) spleen, and (**D**) muscle of WT and *bcl3^−/−^* zebrafish upon Dex treatment. The data was analyzed from three independent experiments performed in triplicate. Statistical significance was assessed using one-way ANOVA followed by Tukey’s post hoc test. *** *p* < 0.001 ** *p* < 0.01, * *p* < 0.05, ns indicates not significant. Error bars represent SD.

We further assessed the effects of Dex treatment on the survival of adult *bcl3^−/−^* zebrafish. At 35 dpf, WT and *bcl3^−/−^* zebrafish were injected with Dex every three days. All *bcl3^−/−^* zebrafish in the DMSO treated control group died at 98 dpf; while those *bcl3^−/−^* fish in the Dex treatment group had an 11% survival rate until 138 dpf (Figure 7A). The treatment of Dex partially rescued the survival rate of adult *bcl3^−/−^* zebrafish compared to the DMSO control. *bcl3^−/−^* zebrafish were also sampled at 87 dpf, and Dex treatment was found to have no effects on either the body morphology, or the reduced body length and weight characteristic of the fish (Figure S6). In the liver, spleen, and muscle of those survived *bcl3^−/−^* zebrafish upon Dex treatment >100 dpf, pro-inflammatory cytokines *il1b*, *il6*, *il8*, and *tnfa* were all significantly downregulated compared to the control fish treated with DMSO; however, the expression levels of these genes were still higher than those in the WT zebrafish (Figure 7B,D). In this experiment, the treatment of Dex started at 35 dpf which coincided with the maturation of adaptive immunity in zebrafish (~4–6 weeks post-fertilization) [34,35,36,39]. At this stage, the innate and adaptive immune systems increasingly interact; therefore, the anti-inflammatory interventions might not be able to fully suppress the established inflammatory circuits. Collectively, these results demonstrated that Dex treatment successfully attenuates hyperinflammation and improves survival of *bcl3^−/−^* zebrafish. However, Dex treatment could not restore growth impairments completely in the adult fish which suggested that Bcl3 might have additional physiological functions other than immune regulation. Further investigation into the precise mechanism underlying Bcl3-dependent immune and developmental regulation is needed in the future.

## 4. Discussion

The mammalian Bcl3 protein is an important regulator modulating the transcriptional activities of NF-κB. The dysregulation of its activity has been shown to be associated with a variety of immune diseases, including multiple sclerosis [40,41] and rheumatoid arthritis [42]. This study explores the physiological function of Bcl3 in immune homeostasis using a zebrafish model. *bcl3^−/−^* zebrafish were found to have severe phenotypes including significantly reduced growth and survival rate (Figure 1). None of the NF-κB and IκB family proteins have been shown to have a massive dying phenotype in previous studies using either mice or zebrafish models. In mice, RelA (p65) or IκBα knockouts die at embryonic or neonatal stages from liver failure or hyper-inflammation [43,44]; p50, c-Rel, or p52 knockouts are immunodeficient but viable [45,46,47]; RelB mutants succumb early to severe inflammation [48]. In zebrafish, all five mammalian NF-κB have been identified, and functional analyses demonstrated that these genes are transcriptionally inducible and involved in both developmental and immune processes (Table S1) [25,49,50,51,52,53]. Among zebrafish IκB proteins, fish with a dominant-negative form of IκBα resulted in a shortened body length and the absence of tail formation [25]; IκBβ and IκBε are transcriptionally upregulated upon injury or infection [28,52]. Collectively, these findings confirm that the NF-κB/IκB regulatory network is evolutionarily conserved in zebrafish, though none of the reported mutants exhibit the progressive mortality phenotype observed in *bcl3^−/−^* fish.

RT-qPCR analyses demonstrated that the expression levels of NF-κB subunits remained unchanged in *bcl3^−/−^* zebrafish. Bcl3 modulates NF-κB p50 and p52 transcriptional activity at the post-translational or co-regulatory level. In addition, Bcl3 has been reported to interact with other transcriptional regulators [54] and metabolic pathways [20,55], raising the possibility that Bcl3 influences inflammatory homeostasis through multiple signaling pathways. Therefore, the severe inflammatory phenotype observed in *bcl3^−/−^* zebrafish likely reflects a combination of disrupted NF-κB regulatory dynamics and additional pathways that remain to be characterized.

The absence of thymic Hassall bodies and reduced expression of immune-cell lineage markers indicate impaired T and B-cell development and myeloid homeostasis in *bcl3^−/−^* zebrafish (Figure 3). Hepatic nuclear pyknosis reflects compromised metabolic integrity resulted from *bcl3* deficiency in fish. Transcriptome analyses further revealed the upregulation of various cytokines and chemokines in *bcl3^−/−^* zebrafish (Figure 4); and factors in JAK/STAT-signaling pathway were also found to be upregulated (Figure S4), which may play an essential role in the hyperinflammation. Our zebrafish model allows the study of inflammation in an intact organism, revealing an evolutionarily conserved function of Bcl3 in preventing immune hyperactivation and maintaining organismal homeostasis. Further investigation is needed to identify potential interacting partner protein(s) of Bcl3, and the detailed mechanism of Bcl3-dependent signaling pathway(s) in zebrafish remains to be elucidated.

The anti-inflammatory drug, Dex, treatment successfully prolonged the survival of *bcl3^−/−^* zebrafish which suggested the severe death phenotype is potentially caused by an overactive immune response. In larval stage, zebrafish rely exclusively on innate immunity, and the treatment of Dex completely rescued the death caused by bacterial LPS challenge in *bcl3^−/−^* fish. However, the treatment of Dex in *bcl3^−/−^* adult fish only partially rescued its survival suggested a transition from reversible innate-immune activation in larvae to a persistent adaptive immune activation and metabolic disturbance in adult, which might be linked with impaired steroid biosynthesis and receptor responsiveness revealed in the transcriptomic analyses. In this study, mixed-sex adult zebrafish were used since male and female *bcl3^−/−^* fish displayed highly similar phenotypes in growth, immune dysregulation, and survival. Although no sex-biased differences were observed, we acknowledge that subtle sex-specific effects cannot be fully excluded and consider this a limitation of the study.

Overall, our findings identified Bcl3 as a key regulator essential for maintaining immune homeostatic and promoting organismal survival. This work using a zebrafish model highlighted the protective and anti-inflammatory role of Bcl3, which is a complement to the mammalian findings where the overexpression of Bcl3 drives chronic inflammation and cancer progression.

## Data Availability

The original contributions presented in this study are included in the article/Supplementary Materials. Further inquiries can be directed to the corresponding author(s). The RNA-seq data have been deposited in the GenBank under the BioProject No. PRJNA1372900.

## Supplementary Materials

The following supporting information can be downloaded at: https://www.mdpi.com/article/10.3390/cells14241935/s1, Figure S1: Generation of *bcl3^−/−^* zebrafish using CRISPR/Cas9. Figure S2. Expression of NF-κB subunits in *bcl3^+/+^* and *bcl3^−/−^* zebrafish. Figure S3. Standard curve of food intake assay. Figure S4. Heatmap showing upregulation of the JAK/STAT signaling pathway in *bcl3^−/−^* zebrafish. Figure S5. Basal expression of pro-inflammatory and antiviral genes in *bcl3^−/−^* larvae. Figure S6. Effects of Dex treatment in *bcl3^−/−^* zebrafish. Table S1. NF-κB and IκB family proteins in Mammal vs. Zebrafish. Table S2. Summary of mortality and morphological phenotypes observed in WT and *bcl3^−/−^* zebrafish larvae upon LPS or poly(I:C) stimulation. Table S3. Primer used for CRISPR, HRMA and RT-qPCR.
