# Supplementary material for: Bcl3 Deficiency Leads to Hyperinflammation in Zebrafish
Source: Cells. 2025 Dec 5;14(24):1935. doi: 10.3390/cells14241935 (PMC12730859; doi:10.3390/cells14241935)
Supplement: Supplementary file 1 [file cells-14-01935-s001.zip › cells-3984941-supplementary.pdf]

Supplemental Information

Bcl3 deficiency leads to hyperinflammation in Zebrafish

Chengjian Fan, Nana Ai, Wei Ge, and Vivien Ya-Fan Wang

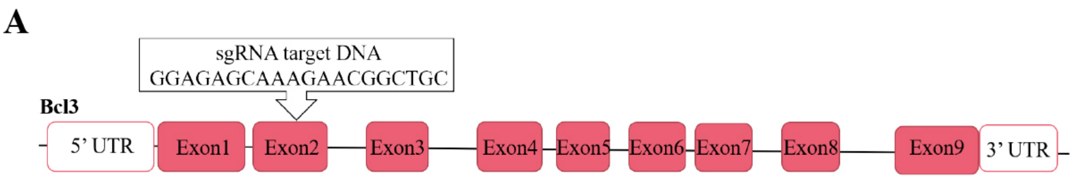

The proportion of *bcl3*$^{+/+}$, *bcl3*$^{+/-}$ and *bcl3*$^{-/-}$ zebrafish in different mutantion type

bcl3 mutant type	*bcl3*$^{+/+}$ number (ratio)	*bcl3*$^{+/-}$ number (ratio)	*bcl3*$^{-/-}$ number (ratio)	Total number of fish
10-bp deletion	17 (30.4%)	32 (57.1%)	7 (12.5%)	56
5-bp deletion	21 (32.8%)	36 (56.3%)	7 (10.9%)	64
7-bp deletion	20 (32.8%)	35 (57.4%)	6 (9.8%)	61

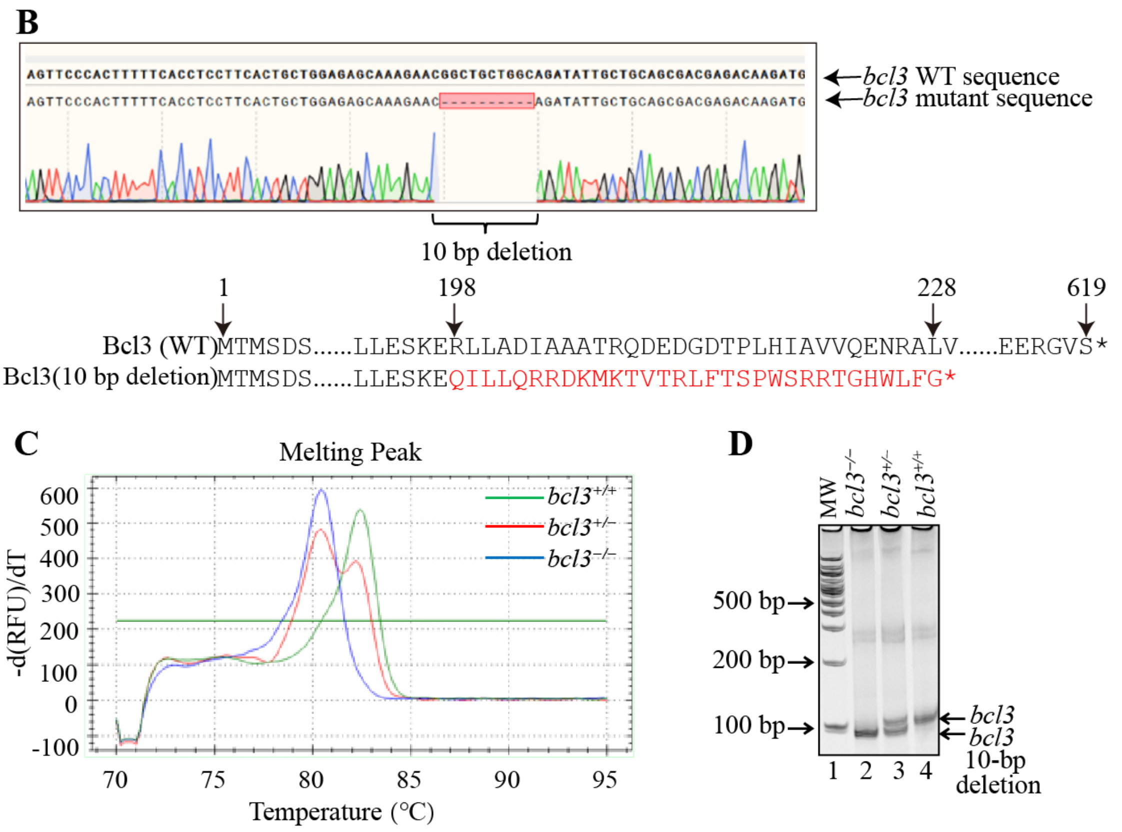

Figure S1. Generation of *bcl3*$^{-/-}$ zebrafish using CRISPR/Cas9. (A) Top: a schematic diagram of zebrafish *bcl3* gene showing nine exons and the sgRNA target site located in exon 2. Bottom: three different *bcl3* deletions,10-, 5- and 7-bp, were generated using the sgRNA; and genotype ratio of 4-month-old zebrafish carrying the three *bcl3* deletions. (B) Top: sequencing chromatograms of *bcl3* mutants reveals a 10-bp deletion in exon 2. Bottom: Bcl3 protein sequence indicates a premature stop codon (*) resulting from the 10-bp deletion. (C) High-resolution melting (HRM) analysis of *bcl3* genotypes. The y-axis represents the negative derivative of fluorescence [-d(RFU)/dT], where RFU denotes relative fluorescence units and reflects the rate of change in fluorescence with

temperature. Distinct melting peaks correspond to *bcl3*$^{+/+}$ (green), *bcl3*$^{+/-}$ (red), and *bcl3*$^{-/-}$ (blue) genotypes. (D) Heteroduplex mobility assay (HMA) confirms distinct band patterns of three *bcl3* genotypes.

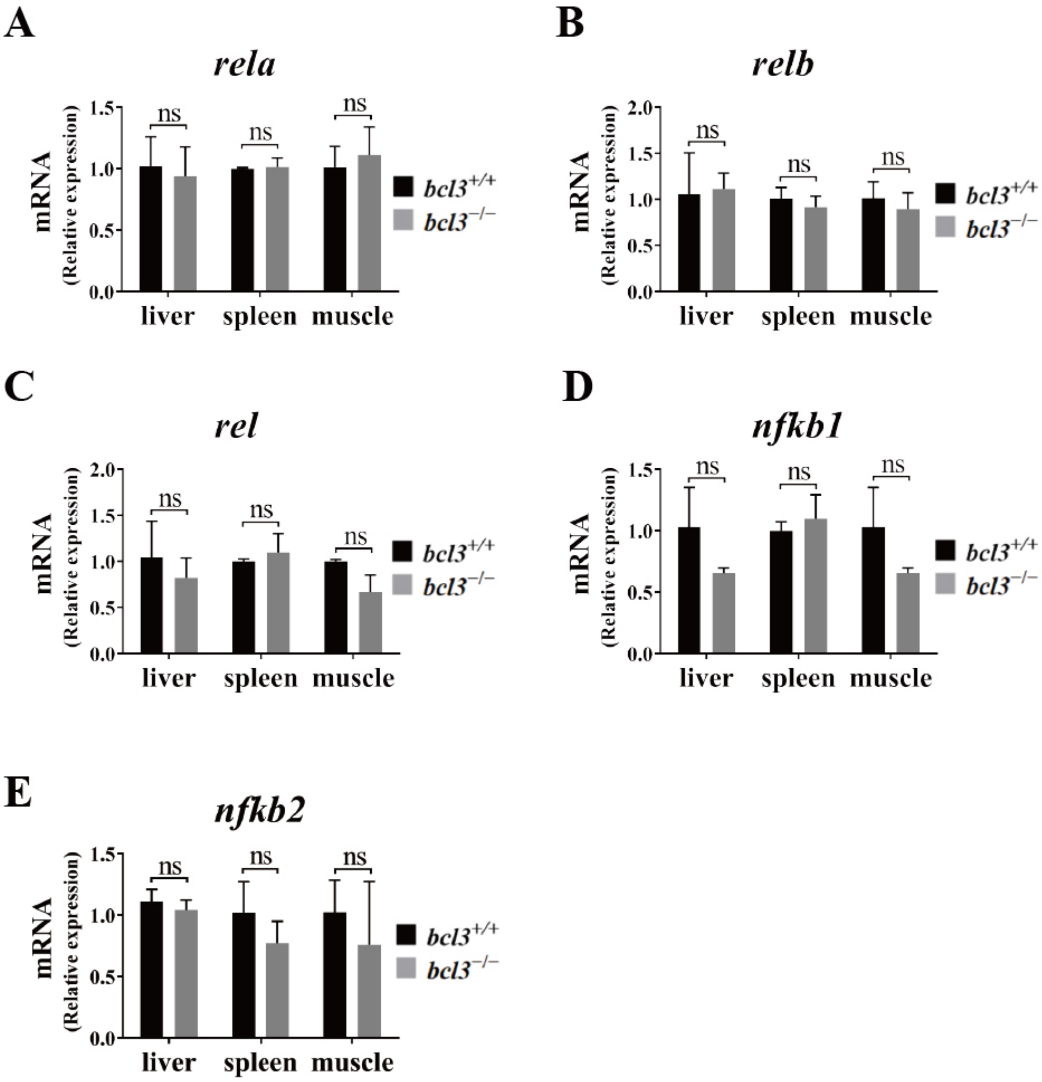

Figure S2. Expression of NF-κB subunits in *bcl3*$^{+/+}$ and *bcl3*$^{-/-}$ zebrafish. Relative mRNA expression of (A) *rela*, (B) *relb*, (C) *rel*, (D) *nfkb1,* and (E) *nfkb2* in liver, spleen and muscle of *bcl3*$^{+/+}$ and *bcl3*$^{-/-}$ zebrafish. No significant (t-test) differences were detected in the expression of any NF-κB subunits between genotypes.

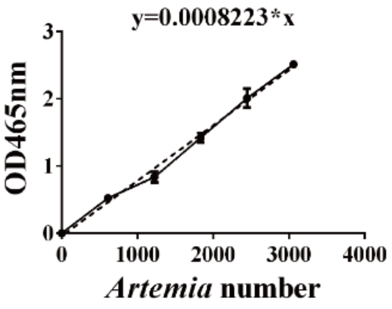

Figure S3. Standard curve of food intake assay. A standard curve showing the relationship between the number of *Artemia* (x-axis) and optical density (OD) (y-axis).

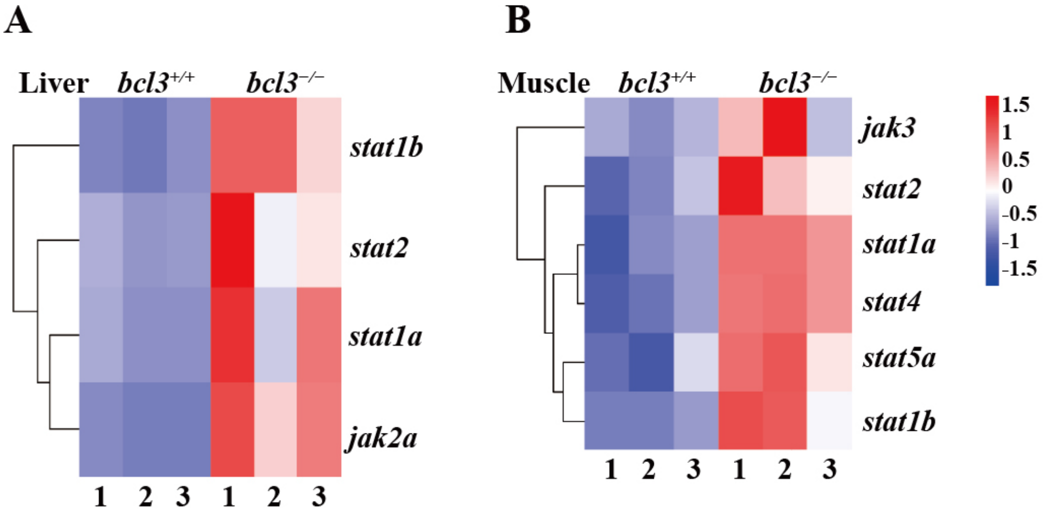

Figure S4. Heatmap showing upregulation of the JAK/STAT signaling pathway in *bcl3*$^{-/-}$ zebrafish. RNA-seq heatmaps displaying normalized expression (Z-scores) of JAK/STAT pathway genes in (A) liver and (B) muscle of *bcl3*$^{-/-}$ zebrafish compared with *bcl3*$^{+/+}$ controls. Color intensity represents relative expression. Differential expression significance (padj < 0.05).

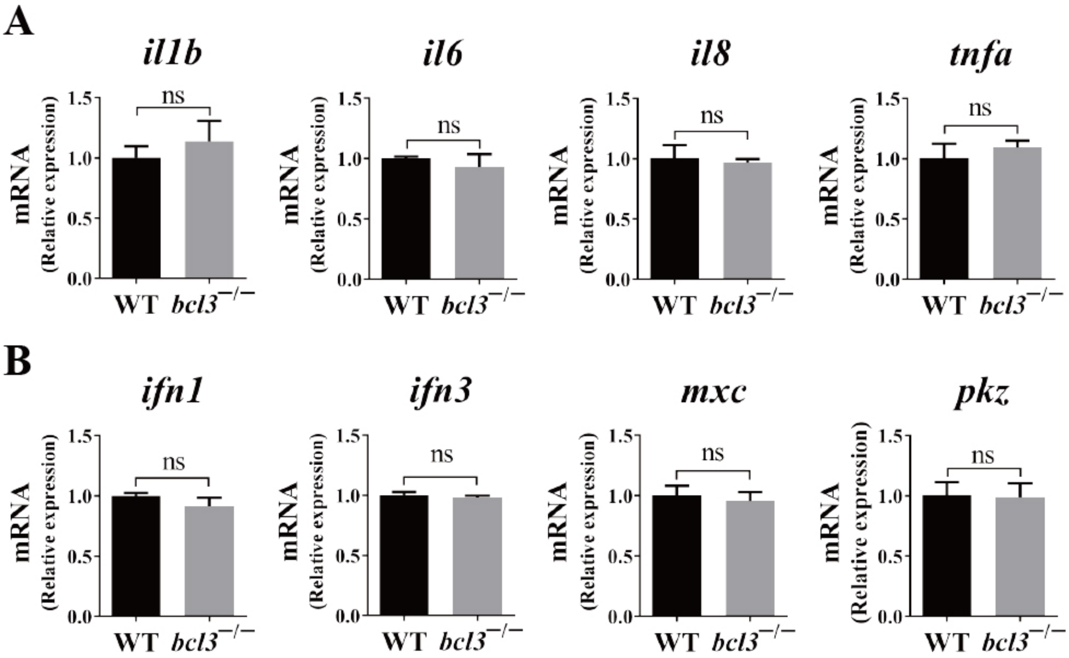

Figure S5. Basal expression of pro-inflammatory and antiviral genes in *bcl3*$^{-/-}$ larvae. RT-qPCR analysis of (A) pro-inflammatory genes *il1b*, *il6*, *il8* and *tnfa*, and (B) antiviral genes *ifn1*, *ifn3*, *mxc*, and *pkz* in 4-day-old *bcl3*$^{-/-}$ and WT larvae.

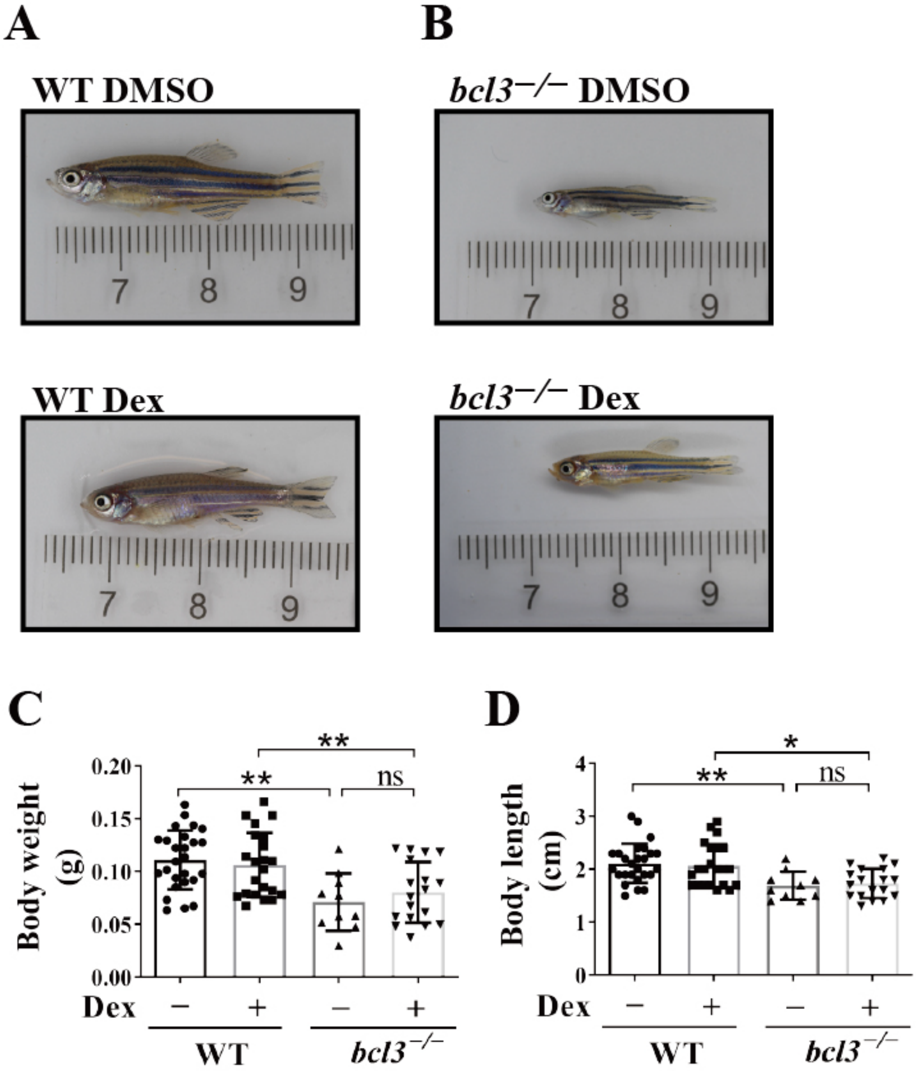

Figure S6. Effects of Dex treatment in *bcl3*$^{-/-}$ zebrafish. Representative images of (A) WT and (B) *bcl3*$^{-/-}$ zebrafish upon Dex treatment. Pictures were taken at 87 dpf. (C) Body weight and (D) body length of WT and *bcl3*$^{-/-}$ zebrafish upon Dex treatment. DMSO was used as a control. Statistical significance was assessed using one-way ANOVA followed by Tukey's post hoc test. $^{**}p<0.01$, $^{*}p<0.05$, ns indicate not significant. Error bars indicate SD.

Table S1. NF-κB and IκB family proteins in Mammal vs. Zebrafish

	Protein	Mammal	Zebrafish	Sequence similarity (%)	Functions in zebrafish studies
NF-κB	RelA (p65)	√	√	55.76	*rela*$^{-/-}$ embryo disturbs mesoderm development, apoptosis, viral immune response [22].
	RelB	√	√	41.27	Zebrafish RelB sequence was submitted in 2003 (ZFIN ID: ZDB-GENE-030131-9531).
	cRel	√	√	50.37	Zebrafish Rel has weak binding affinity to DNA [41]. Upregulated upon bacterial and viral infections [42,43].
	NF-κB1 (p105/p50)	√	√	59.31	Injury in zebrafish leads to upregulation of *nfkb1* expression [44].
	NF-κB2 (p100/p52)	√	√	49.54	*p100 + p65 morpholino,* embryo lacks caudal region [20].<br>*nfkb2* acts as a key regulator of pro-inflammatory and tumor-related genes in zebrafish [45].
IκB	IκBα	√	√	IκBα-a: 50.33<br>IκBα-b: 49.51	Embryos expressing dominant-negative mIκBαM have shorter body length and smaller or absent tails [20].
	IκBβ	√	√	38.11	Injury in zebrafish leads to upregulation of *nfkbib* expression [44].
	IκBε	√	√	43.88	*nfkbie* transcription is upregulated in brain injury [44].
	IκBγ(p105)	√	√	59.31	-
	IκBδ(p100)	√	√	49.54	-
	Bcl3	√	√	43.38	Zebrafish Bcl3 sequence was submitted to database in 2006 (ZFIN ID: ZDB-GENE-061013-1).<br>*bcl3*$^{-/-}$ resulted in reduced growth and survival (*this study*).
	IκBζ	√	-	-	-
	IκBNS	√	-	-	-

Table S2. Summary of mortality and morphological phenotypes observed in WT and *bcl3*$^{-/-}$ zebrafish larvae upon LPS or poly(I:C) stimulation.

	LPS injection Larvae number (rate)			Poly(I:C) injection Larvae number (rate)		
	WT	*bcl3*$^{-/-}$	Significance	WT	*bcl3*$^{-/-}$	Significance
Total number of injected larvae	400	310	-	350	218	-
Death	23 (5.8%)	43 (13.9%)	***	18 (5.1%)	23 (10.6%)	*
Phenotype: total number (rate)	135 (33.8%)	192 (61.9%)	***	87 (24.9%)	91 (41.7%)	***
• Pericardium edema	44 (11%)	87 (28.6%)	***	31 (8.9%)	13 (6%)	ns
• Spine curvatures	22 (5.5%)	35 (11.3%)	***	17 (4.9%)	43 (19.7%)	***
• Uninflated swim bladder	87 (21.8%)	95 (30.7%)	-	34 (9.7%)	32 (14.7%)	-
• Swim bladder loss	75 (18.8%)	72 (23.2%)	-	16 (4.6%)	17 (7.8%)	-
• Swelling	54 (13.5%)	140 (45.2%)	***	44 (12.6%)	27 (12.4%)	ns
• Necrotic yolk	68 (17%)	113 (36.5%)	***	32 (9.1%)	40 (18.4%)	***

Binary outcomes (presence/absence of mortality or morphological abnormalities) were analyzed using chi-square tests. * $p < 0.05$, *** $p < 0.001$, ns indicates not significant. “Uninflated swim bladder” and “Swim bladder loss” represent mutually exclusive states of the same organ; therefore, separate statistical comparisons between these two categories were not performed, and swim bladder abnormalities are instead represented within the “total phenotype” category.

Table S3. Primer used for CRISPR, HRMA and RT-qPCR.

Gene	Primer name	Sequence (5’→3’)	Application
bcl3	sgRNA	TAGGAGAGCAAAGAACGGCTGC	CRISPR
	sgRNA	AAACGCAGCCGTTCTTTGCTCT	
	Bcl3-exon2-F	ATGCACATGCCAGTTCCCAC	HRMA
	Bcl3-exon2-R	CGTCTTCATCTTGTCTCGTCG	
ef1a	*ef1a*-F	CCAAGGAAGTCAGCGCATAC	RT-qPCR
	ef1a-R	CCTCCTTGCGCTCAATCTTC	
rag2	zqrag2-F	GATGGAGAAGCTACGGCACA	
	zqrag2-R	TCTCGGCCAAAGTACAGCTC	
mpeg1	zqmpeg1-F	TCCACCGTTCACAAGACCAC	
	zqmpeg1-R	CTCTCACCCAAGAACGCTCA	
mpx	zqmpx-F	TTCCAGAAAATCCGAGATGG	
	zqmpx-R	ACACAGAGGCCAGAGCTGTT	
cd79a	zqCD79a-F	GGCTGGTGCTTTTCTTGTCTT	
	zqCD79a-R	GAGACCTGCACACGCAGTAA	
il1b	zqIL1b-F	TGGACTTCGCAGCACAAAATG	
	zqIL1b-R	GTTCACTTCACGCTCTTGGATG	
il6	zqIL6-F	CCGAAATATCTGGAGACGAAGT	
	zqIL6-R	GGTCTGAAGGTTTGAGGAGAG	
il8	zqIL8-F	GAATGAGCTTGAGAGGTCTGG	
	zqIL8-R	GATCTTCTTAACCCATGGAGCA	
il10	zqIL10F	TTTGTGGAGGGCTTTCCTTTA	
	zqIL10-R	TGAAGAGCTGTTGGCAGAAT	
tnfa	zqTNFa-F	GCTGGATCTTCAAAGTCGGGTG TA	
	zqTNFa-R	GCTGGATCTTCAAAGTCGGGTG TA	
ifn1	zqIFN1-F	GAGCACATGAACTCGGTGAA	
	zqIFN1-R	TGCGTATCTTGCCACACATT	
ifn3	zqIFN3-F	AGAGAGGCTTCGCTCAAGGTTT	
	zqIFN3-R	TTTTTTAGTATGAACTCCAGGGT GC	
mxc	zqMxC-F	GAGGCTTCACTTGGCAACTC	
	zqMxC-R	TTGTTCCAATAAGGCCAAGC	
pkz	zqpkz-F	GGAGCACCGTACAGGACATT	
	zqpkz-R	CTCGGGCTTTATTTGCTCTG	
rela	zqRela-F	CATTCCCTACGGCTAAACGA	
	zqRela-R	CGGACACTGCTGGTACACTG	
relb	zqRelB-F	CGCATGTCTGATCGGATGGA	
	zqRelB-R	TTCCTCTTGCGGTTCACTCC	
rel	zqcRel-F	CAGTGTGAAGGGAGGCGATG	
	zqcRel-R	CTGAGTGAAGAACCGCACCT	
nfkb1	zqNFkB1-F	TCTGCTCTGCGACAAAGTCC	
	zqNFkB1-R	GAAATCCCCATACGCCTCCC	
nfkb2	zqNFkB2-F	CCCAATTCGCTCAGCCCTAA	
	zqNFkB2-R	AATCCCACACAGCTTGCTCA	
